# Increasing Daily Happiness in Family Caregivers of People Living With Dementia With a Mobile App-Based Program

**DOI:** 10.1093/geroni/igaf122.1138

**Published:** 2025-12-31

**Authors:** Felipe Jain, Paulina Gutierrez-Ramirez, Kimberly Dueck, Maria Galvez, Liliana Ramirez Gomez

**Affiliations:** Massachusetts General Hospital / Harvard Medical School, Boston, Massachusetts, United States; Massachusetts General Hospital, Boston, Massachusetts, United States; Massachusetts General Hospital, Boston, Massachusetts, United States; Massachusetts General Hospital, Boston, Massachusetts, United States; Massachusetts General Hospital, Boston, Massachusetts, United States

## Abstract

Family caregivers of people living with dementia are at high risk for depression and stress. Several caregiver interventions have been found to reduce symptoms of depression and stress on pre-to-post outcomes testing, but few have characterized impacts on day-to-day caregiver symptoms. Mentalizing imagery therapy (MIT) is a mindfulness and guided imagery approach that provides tools to relax and improve understanding of others (including the person living with dementia). Using ecological momentary assessments (EMA), we studied the effects of MIT on day-to-day experiences of happiness and stress of family caregivers of people living with dementia. We conducted an 8-week randomized, controlled trial of caregiver skills (CS) training with and without MIT delivered by the MGH CareDoc mobile application platform. Interventions consisted of mobile applications and optional weekly virtual groups with content tailored toward each group. EMA ratings of happiness and overall stress over the prior 24 hours were obtained daily through CareDoc from days 1 to 56. 110 caregivers were randomized (n = 55 in each arm). 101 completed the trial (with no difference in attrition between groups). Group X Time analyses found that those assigned to CS with MIT exhibited improvements in daily ratings of happiness (p<.0001) and stress (p<.001) relative to the CS only arm. In this randomized, controlled trial, MIT with CS exhibited superior effects to CS alone on improving daily happiness and stress. This study contributes needed data on MIT’s impacts on day-to-day experiences and is among the first to show improvements in daily symptoms in family caregivers.

